# Qualification of an enzyme-linked immunosorbent assay for quantification of anti-Vi IgG in human sera

**DOI:** 10.3389/fimmu.2024.1466869

**Published:** 2024-10-16

**Authors:** Martina Carducci, Luisa Massai, Elisa Lari, Bianca Semplici, Maria Grazia Aruta, Daniele De Simone, Pietro Piu, Emanuele Montomoli, Francesco Berlanda Scorza, Silvia Grappi, Miren Iturriza-Gómara, Rocio Canals, Simona Rondini, Omar Rossi

**Affiliations:** ^1^ GSK Vaccines Institute for Global Health (GVGH) S.r.l., Siena, Italy; ^2^ VisMederi S.r.l., Siena, Italy; ^3^ Department of Molecular and Developmental Medicine, University of Siena, Siena, Italy

**Keywords:** enzyme - linked immunosorbent assay (ELISA), Vi, human, vaccine, enteric fever, typhoid, antibodies

## Abstract

Effective vaccines against *Salmonella* Typhi, targeting the Vi capsular polysaccharide, have been developed and are being introduced into routine immunization in endemic countries. Vi conjugated vaccines are also being tested in new multi-component vaccine formulations. Simple, high-throughput and cost-effective assays to quantify Vi-specific IgG in clinical sera are needed. In this study we present the development and qualification of a new anti-Vi ELISA with continuous readout, which expresses results as ELISA Unit/mL (EU/mL). We have qualified the assay in terms of precision, linearity and specificity, demonstrating performance in line with a commercially available anti-Vi ELISA. We have also calibrated the assay against the 16/138 anti-Vi international standard and established conversion factor between EU/mL and international units/mL, to allow comparability of results across studies. In summary, this new assay met all the suitability criteria and is being used to evaluate anti-Vi responses in clinical studies.

## 1 Introduction

*Salmonella enterica* serovar Typhi is the etiological agent of typhoid fever, causing over 9.2 million cases and 110,000 deaths each year globally (1), particularly in children and young adults living in low and middle income countries (2, 3).

The surface capsular polysaccharide Vi represents a virulence factor and the main protective antigen (4, 5), and is a key target for vaccine development. Over the years, several vaccines have been developed. The first vaccines developed were the oral live attenuated Ty21a vaccine and the injectable plain Vi polysaccharide. However, none of them are effective in children below two years of age and have not been licensed for this age group (6, 7). By conjugating the Vi polysaccharide to carrier proteins, Typhoid Conjugate Vaccines (TCV) have been developed (8–11) to protect children and infants, and three TCV have achieved pre-qualification status by WHO so far (12–14).

Further efforts are currently ongoing to formulate TCV with other vaccine candidates against *Salmonella* Paratyphi A (15, 16), or invasive non-typhoidal salmonellosis (17–19). This combination strategies will broaden the protection against multiple *Salmonella* diseases (17).

Since Vi is the target antigen of all current monovalent TCV and will need to be assessed also in new multivalent combinations, the development of an accurate, high-throughput and cost-effective method to determine the antigen specific IgG levels in sera is critical.

In the past, efforts have been made to establish an international anti-Vi reference standard serum and to optimize immunoassays to measure concentration of anti-Vi IgG. A first freeze dried standard serum, called TYS, was originated from a horse immunized with rough and smooth *S*. Typhi in 1935 (5). Afterwards, several collaborative studies involving multiple labs based in various countries were conducted using sera from vaccinees. In 2010, the standard 10/126 was developed from freeze dried pool of sera from subjects immunized with Vi-TT (20). In 2011, Vi-IgGR1, a purified fraction of IgG from plasma donations by volunteers vaccinated with Vi-rEPA, was quantified to contain 33 μg anti-Vi IgG/mL (10). Both Vi-IgGR1 and 10/126 had similar performance when tested using a commercial human anti-*S*. Typhi Vi IgG ELISA (Binding Site, UK), but results obtained when running ELISA methods developed in different labs testing the same sera were highly variable (20). Thus, a new standard (namely 16/138) was obtained by pooling sera from subjects immunized with Vi and Vi-TT conjugate vaccines (21). In a big collaborative study using various ELISA methods, the 16/138 standard serum passed all control criteria and was selected as WHO international standard with an assigned 100 IU/mL (20). The possibility to access 16/138 enabled assessing the performance and reproducibility of customized and commercial ELISAs across multiple laboratories and compare results across studies.

In this study we present the development of a new ELISA method based on continuous readout to measure anti-Vi IgG antibodies expressed as ELISA Unit/mL (EU/mL). We have characterized the assay in terms of precision, dilution linearity and specificity. We have calibrated the assay against the 16/138 international standard and compared the assay’s performance against a commercially available ELISA kit. Results demonstrated the suitability to use the new anti-Vi ELISA in clinical studies, with the opportunity to express anti-Vi IgG as EU/mL, µg/mL, or IU/mL, therefore enabling comparison across studies.

## 2 Materials and methods

### 2.1 anti-Vi customized ELISA

Anti-Vi specific total IgG were measured in serum samples using Vi from *Citrobacter freundii* (22) (equivalent to the Vi produced by *S*. Typhi (23)) as coating antigen (52-53% acetylation (24)), adapting a method previously described (22, 25, 26). Plates were coated with Vi in PBS at a final concentration of 1 µg/mL overnight (16 h) at 4°C of Maxisorp 96-well round bottom plates (Nunc Maxisorp, 449824), followed by aspiration (without wash) and blocking with 5% PBS milk (Millipore, 70166) for 1 h at 25°C. Plates were then washed 3 times with PBS-Tween 0.05% (Sigma-Aldrich, P1379) (PBS-T), before addition of primary antibodies (sera samples) diluted in 5% PBS milk, and were incubated for 2 h at 25°C. Each human serum sample was run in triplicate at different dilutions (1:100, 1:4000, and 1:160,000, respectively) in PBS milk 5% in different plates. Standard sera made of 10 calibrators two-fold apart diluted in PBS milk 5% plus two blank wells with no sera were run in duplicate on each plate. High control and low control (HC and LC, respectively) were run on each plate at dilution able to give as result a range equivalent to 0.21-0.89 and 0.39-1.73 EU/mL, respectively, for LC and HC. Plates are then washed 3 times with PBS-T and incubated for 1 h at 25°C with alkaline phosphatase goat anti-human IgG (Sigma-Aldrich, A3187) diluted 1:5000 in PBS-T plus 0.1% BSA (Sigma-Aldrich, A9418), before the final 3 washes with PBS-T and addition of p-Nitrophenyl phosphate substrate (Sigma-fast N2770, Sigma-Aldrich), for 1 h at 25°C. Absorbances were read with a spectrophotometer at OD 405 and 490 nm (Biotek automatic plate reader), maintaining strict timing between plates.

ELISA units are expressed in relation to a five-parameter human antigen-specific antibody standard serum curve composed of 10 standard points and 2 blank wells (run in duplicate on each plate). One ELISA unit is defined as the reciprocal of the dilution of the standard serum that gives an absorbance value (optical density measured at 405 nm subtracted to optical density measured at 490 nm—the latter being the background wavelength of the plastic of the plate) equal to 1.

The primary anti-Vi standard serum was calibrated and antigen concentration was set at saturation of the signal. Several QC criteria were applied on each run, in particular, the R-square value for the 5 PL curve fit of the standard dilution series maximum background OD, the minimum value of OD maximum, range of acceptance in terms of OD for 1 EU/mL, deviation to the expected EU/mL both for high and low controls. If at least one of the above-mentioned criteria was not met, the entire layout was repeated under the same experimental conditions. A sample is instead considered valid if the EU/mL determined as average from the values obtained in the three independent plates have a CV% < 30% at the dilution selected for obtaining results (the ones in which OD values obtained fall within the linear part of the standard curve); if not met, the sample was re-run under the same assay conditions (frequency of re-run due to failure of this parameter is <2%).

### 2.2 Anti-Vi ELISA using commercial kit

The VaccZyme ELISA was carried out according to the manufacturer’s instructions (Binding Site). Vi of *S.* Typhi was used as coating antigen. Five calibrators, covering a range from 7.4 to 600 U/mL, with a high and a low positive control included in each run. The assay comes with a sensitivity of 7.4 U/mL. Four independent replicates of serum samples were run at 1:800, 1:1600 or 1:3200 dilutions each tested in triplicates and plates were read at OD_450nm_. Results were obtained by interpolating the OD_450nm_ values against the standard curve (as per manufacturer instructions) and were represented by the average U/mL of the triplicate dilution falling within the quantifiable range of the curve, taking into account the dilution factor.

### 2.3 Sample preparation to assess precision, linearity, limit of quantification, and specificity

Vi Human Standard Serum and five samples with different IgG concentrations have been generated by pooling sera from high responder subjects originally enrolled in Vi-CRM_197_ (TCV) Phase 1 trial in Belgium (25). Working aliquots of the standard sera were stored at −80°C until use. Human IgG-depleted serum (Molecular Innovations cod. HPLA-SER-GF) was used as a negative matrix.

Samples used to assess standard curve accuracy: Vi Human Standard Serum has been used to prepare 24 standard curves composed of 10 two-fold dilutions starting at 10 EU/mL and including 2 blanks.

Samples used to assess precision, and dilution linearity: Vi Human Standard Serum neat or diluted ½, ¼, 1/8, 1/16, 1/32, 1/64 in negative matrix (IgG depleted sera from molecular diagnostic) were assayed independently in a standard assay by two operators working on the same days, in four independent replicates on each plate, on three different days (24 measurements in total for each individual serum).

Samples to assess specificity: human standard sera were preincubated overnight at 4°C with an equal volume of homologous competitor (Vi) at the final concentrations of 50, 20, 5, 1, 0.1 and 0.01 µg/mL in PBS + 5% milk, in comparison to sera diluted overnight 1:100 in PBS + 5% milk (negative control). The lowest concentration of Vi able to cause a reduction of the ELISA Units ≥ 80% was then used to determine the homologous and heterologous specificity, assessed with samples incubated with OAg from a different *Salmonella* species (*S.* Typhimurium) and a different pathogen (*Shigella sonnei*) in comparison to undepleted control. All samples were incubated overnight (16–18 h) at 4°C prior to being tested.

### 2.4 Calculations and statistical analysis

Test results have been analyzed using Excel, GraphPad PRISM software version 7 and Minitab 18 (Minitab Inc., Chicago, IL, USA). Geometric and arithmetic mean, standard deviation, and coefficient of variation were the major statistics.

To calculate the standard curve accuracy, percentage Residual Error RE% ((recalculated value-nominal value)/nominal value*100 (back calculated error)) was plotted in function of the nominal concentration. Lower and upper limit of standard curve accuracy (LLSCA and ULSCA, respectively) were set at the nominal value of the standard curve at the last and the first dilutions, respectively, with a 90% prediction interval of RE% within the acceptance range of [-25%; 25%].

Linearity was assessed by plotting resulting titers (EU/mL after multiplication per dilution factor) divided by the median of all results vs each dilution with fitted model and 95% CI with acceptance criteria on each datapoint to fit on the 0.7-1.3 range. In the dilution linearity and precision analysis eight outliers were excluded using the Hampel filter methods (all found in the highest dilution of tested samples). The Hampel filter method consists of considering outliers the values outside the interval I=M−k∗b∗MAD; M+k∗b∗MAD equivalent to [19.84, 2019.53] where MAD (Median Absolute Deviation) is defined as the median of the absolute deviations from the median M, b is a constant linked to the distribution of the data and the factor k was set to 2. Linearity of the assay has been determined, in addition, by calculating log2-transformed GM (Geometric Mean) plotted against log2-transformed samples dilution and the coefficient of determination (R2) of the regression and 95% confidence interval of the slope.

Precision was assessed by calculating inter and intraassay precision using Minitab; ANOVA with variance component analysis (mixed effect model with random factors) was used to estimate the intermediate precision, and to evaluate the contributions of the operator and day of analysis to the variability. Acceptance criteria were set at CV% resulted ≤ 20% for intra-assay precision and ≤ 25% for inter-assay precision.

The specificity was expressed as % of inhibition, defined as = (EU/mL Control – EU/mL Inhibited sample)/EU/mL control x 100.

## 3 Results

### 3.1 Anti-Vi ELISA setup and performance assessment

**Figure 1:**
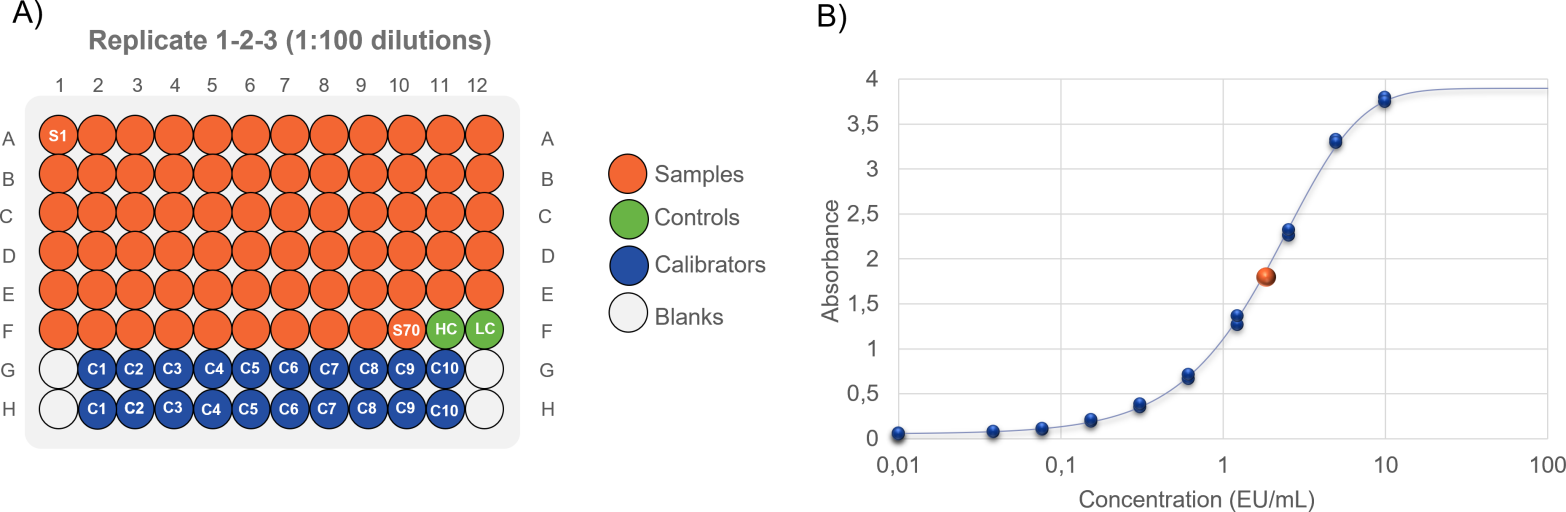
**(A)** ELISA layout: 1:100 diluted samples are assayed in triplicates on different plates. Up to 70 samples can be tested on the same layout. If necessary, the same layout can be assessed 1:4000 and 1:160000 fold diluted, making 1:40-fold serial dilution from the previous one. One sample layout can result from three to nine 96 well plates. **(B)** Sigmoidal Logistic 5PL interpolation of sample on the standard curve.

We developed and qualified a new ELISA method to quantify anti-Vi IgG in human samples, based on a method we previously used for preclinical and clinical testing (22, 26–30). In this assay, one ELISA unit (EU) is defined as the reciprocal of the dilution of the standard serum that gives an absorbance value equal to 1. Each plate contains high and low control sera with assigned EU/mL, a calibrated standard curve composed of 10 serial dilution points (in duplicate), and 4 blank wells. Individual samples are tested in triplicates at up to three dilutions on different plates. EU/mL of each sample are obtained by interpolating the OD values against the standard curve run on each plate. By using this method, up to 70 different sera can be assayed on a set composed of nine 96-well plates (**Figure 1**).

To be valid, an assay must pass multiple non-mutually exclusive quality control criteria (R-square value for the 5 PL curve fit of the standard dilution series > 0.98, maximum background OD < 0.15, the minimum value of OD maximum >2.6, with 0-5-2 range of acceptance in terms of OD for 1 EU/mL, deviation to the expected EU/mL both for high and low controls <40%).

**Figure 2:**
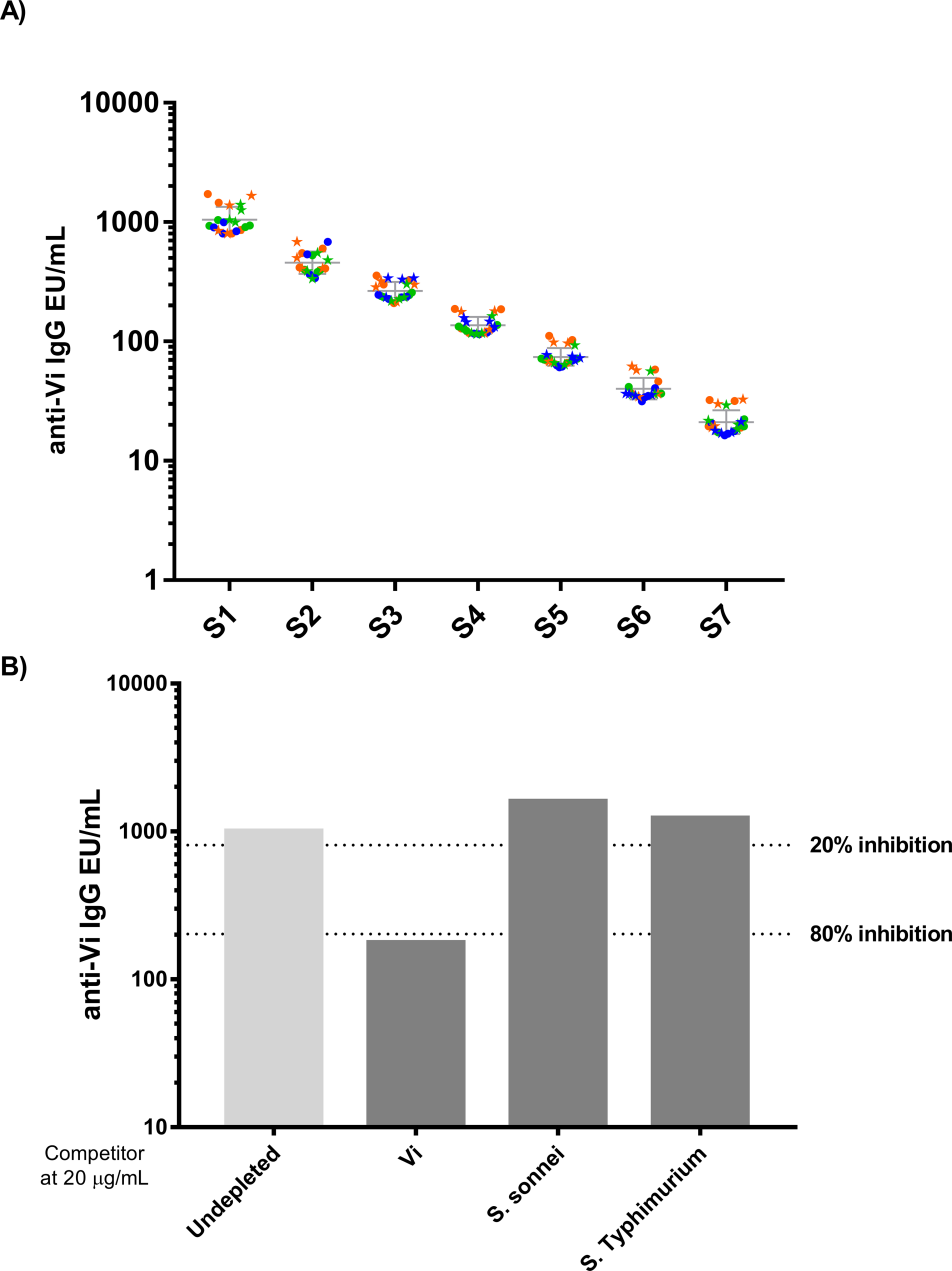
ELISA Assay Parameters **(A)** ELISA precision and dilution linearity. A total of 24 repeated measurements of EU/mL from independently handled samples, by two operators on three different days. Single repeats of each operator are represented by circles symbols (for operator 1) and star symbols (for operator 2), repeats on different days are shown in orange for day 1, green for day 2 and blue for day 3. Geometric means and geometric standard deviations from 24 repeats are represented by the grey line for each of the tested samples. **(B)** Homologous and Heterologous specificity.

When we tested the accuracy of the standard curve according to this ELISA setup, we measured an accuracy range between 0.113 EU/mL and 4.456 EU/mL. To assess linearity and precision, the anti-Vi reference serum, obtained by pooling high responder vaccine sera, was tested undiluted and after six sequential dilution steps (2-fold apart) in 4 independent replicates, run on 3 days and by 2 operators (**Figure 2A**). The assay proved to be linear, either in terms of linearity graphs, which fell within 0.7-1.3 predefined range, and as per regression analysis (R^2 =^ 0.9917). The quantification of the seven samples in the different replicates provided an intra-assay precision with CV% ≤ 20% (7.32 to 17.05%) and inter-assay precision with a CV% ≤ 25% (7.11 to 15.88%). These values are within the limits of acceptability that were predetermined for this parameter.

To assess the specificity, an initial set-up experiment was performed, by pre-incubating homologous purified Vi antigen at different concentrations with test samples to determine the lowest concentration of Vi able to cause a reduction of the ELISA Units of ≥ 80%. This was shown to be 20 µg/mL of Vi. Such concentration was used to assess the homologous and heterologous specificity, with Vi used as homologous competitor and <i>Shigella sonnei and S. Typhimurium OAg used as heterologous competitors. The assay resulted to be specific: a signal’s inhibition >80% (82.37%) was observed with Vi, while no inhibition was found with the heterologous antigens (**Figure 2B**).

### 3.2 Calibration against the international anti-Vi standard

**Figure 3:**
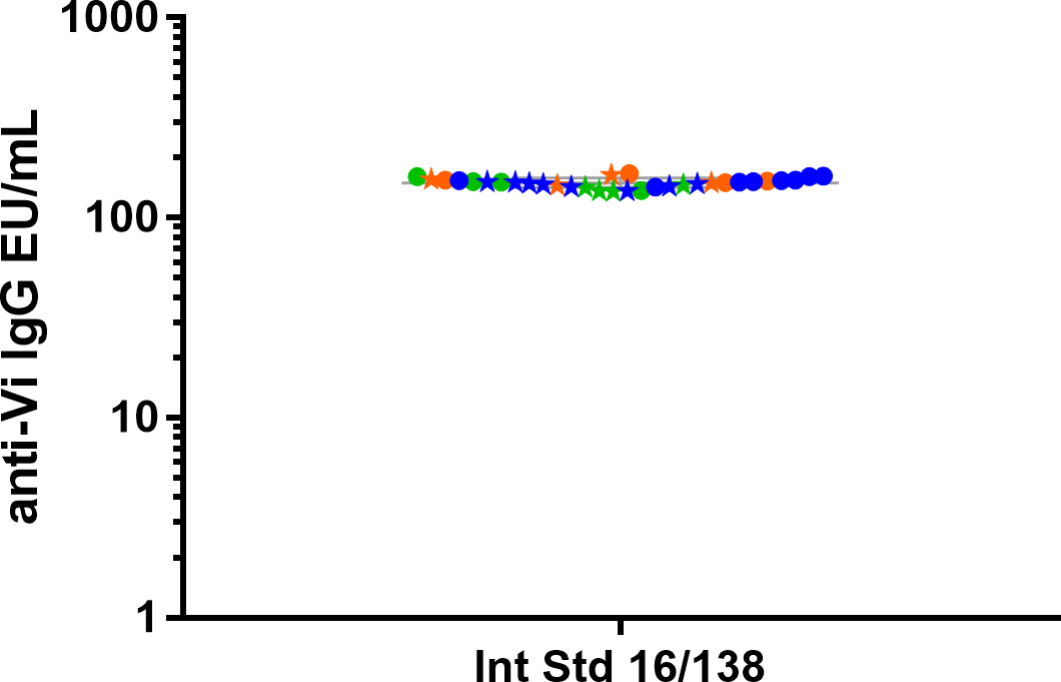
Anti-Vi polysaccharide IgG WHO international standard (16/138) was tested in 32 independent replicates by two operators (circles and stars for operator 1 and 2, respectively) in three different days (repeats of Days 1 (orange) and 2 (green) are 4, while in Day 3 (blue) they are 8). Geometric means and geometric standard deviations from 24 repeats are represented by the grey line for each of the tested samples.

We calibrated the assay against the international standard to establish a conversion factor between EU/mL and International Unit/mL (IU/mL). To this end, anti-Vi IgG international standard (16/138), was assayed for a total of 32 independent tests, run in four replicates during four different days, by two operators (**Figure 3**). The Intermediate Precision and Intra-Assay Precision of these tests were 4.09% and 4.82%, respectively.

The conversion factor between EU/mL and IU/mL was therefore calculated as the ratio between the average EU/mL obtained considering all the replicates reported in **Figure 3** (equal to 148.04 EU/mL, range 132.46-163.62 CI95%) and the 100 IU/mL assigned to 16/138, and thus resulted to be 0.676 (**Table 1**). The IgG values, expressed as EU/mL, can therefore be converted to µg/ml based on the conversion factor between EU/mL and IU/mL (0.676) and the one previously established between IU/mL and µg/mL (0.01775 (10)), thus resulting in 1 EU/mL equivalent to 0.137 µg/mL of anti-Vi IgG (**Table 1**).

**Table 1:** *According to Rijpkema S, 2018 (20) 163 IU/mL can be assigned to Vi-IgGR1, 2011, which contains 33 µg/mL of anti-Vi Antibodies (10).

	Average (EU/mL)	IU/mL	Conversion Factor EU/mL to IU/mL	Conversion Factor IU/mL µg/mL*	Conversion Factor EU/mL to µg/mL
**Int Std**	148.04	100	0.676	0.202	0.137

### 3.3 Comparison of customized and commercial ELISA

**Figure 4:**
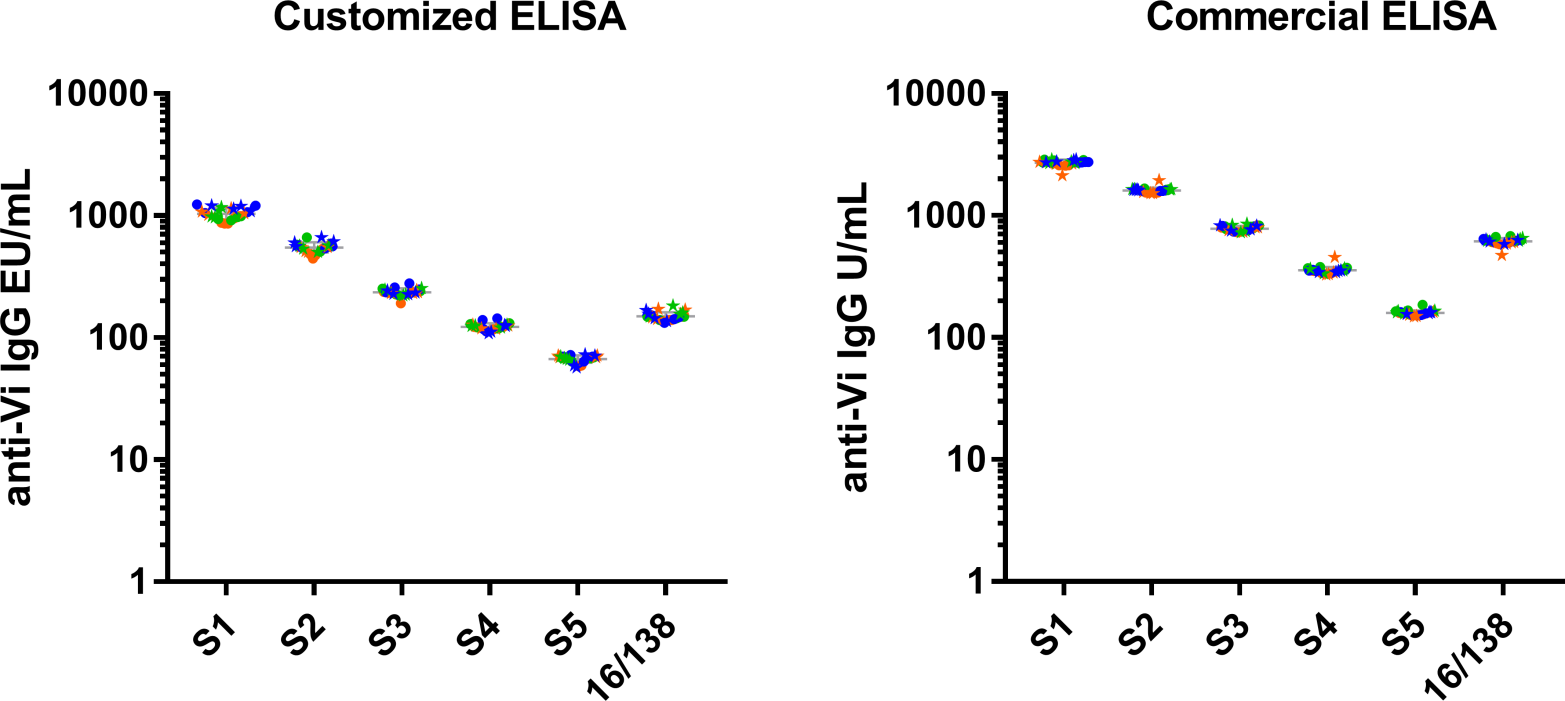
Commercial ELISA vs customised ELISA. A total of 24 repeated measurements of EU/mL from single independently handled samples, by two operators on three different days. Single repeats of each operator are represented by circle symbols (for operator 1) and star symbols (for operator 2). Repeats on different days are shown in orange for day 1, green for day 2 and blue for day 3. On each assay results are expressed in their respective arbitrary units (EU/mL for customized ELISA and U/mL for commercial ELISA). Geometric means and geometric standard deviations from 24 repeats are represented by the grey line for each of the tested samples.

The qualified ELISA was compared with an anti-Vi ELISA commercial kit used in the field (9, 10, 20, 25, 31–33). This was done by testing five different pooled serum samples with different anti-Vi IgG content (1037, 548, 236, 123 and 67 EU/mL for S1, S2, S3, S4 and S5 respectively) and the 16/138 standard serum, in three different days, by two operators (**Figure 4**).

Both assays resulted to be precise, with CV% reproducibility and CV% repeatability, respectively, between 4.85-7.40 and 3.30-6.56 for the commercial ELISA, and between 6.48-12.76 and 6.35-7.05 for the qualified ELISA. Hence, both assays met the acceptance criteria for repeatability (20%) and reproducibility (25%), confirming their precision. Furthermore, neither the day nor the operator influenced the overall variability in both assays (p-values >0.05 for all the samples).

## 4 Discussion

Vi conjugates represent very effective vaccines against *Salmonella* Typhi and are incrementally being introduced in endemic countries. Several assays to measure anti-Vi antibodies have been used, and the generation of anti-Vi reference standard sera has opened the possibility to compare results from different clinical studies.

In this study we have presented the development of a customized ELISA method and showed its performance. We demonstrated that the assay is precise, linear and specific. The assay can accommodate 70 individual samples in one run of nine 96-well plates, and up to 140 individual samples that can be run by a single operator per day manually. We have also used this assay to test preclinical sera in 384-well plates (30, 34, 35), and have achieved a throughput of 384 individual samples per operator per day. The miniaturization of the assay further reduced the costs of the assay per sample of about 25%.

Previous reports showed that Vi performed worse as coating antigen than poli-L-Vi or biotinylated-Vi (36). However, in our hands, Vi performed well as coating agent and allowed a satisfactory assay performance, similar the one of the commercial kit and in line with the precision envisaged for clinical immunoassays. In addition, the use of non-derivatised Vi for coating offers the advantage of not incurring in risk of modifying important epitopes.

The assay presented here is economically more convenient than the commercial alternative (4 euro/sample versus 50 euro/sample in the 96-well plate format presented here), while performing comparably. In addition, the customized assay relies on standard laboratory plasticware and on an enzymatic reaction based on alkaline phosphatase/para-nitrophenol, which can be easily bridged in case of discontinuation in supply of any specific reagent. This would not be the case if the commercial kit happens to be discontinued.

Finally, taking advantage of the anti-Vi international standard, we have calibrated our assay against 16/138, establishing conversion factors to IU/mL, as well as to µg/mL (10). This offers the possibility to compare results across multiple studies and present them using different units of measure.

In summary, the ELISA method presented in this study represents a valid alternative for the commercial kit and is currently being used to evaluate anti-Vi IgG in human sera.

## Data Availability

The original contributions presented in the study are included in the article/supplementary material. Further inquiries can be directed to the corresponding author.

## References

[1] Hancuh M Walldorf J Minta AA Tevi-Benissan C Christian KA Nedelec Y. Typhoid fever surveillance, incidence estimates, and progress toward typhoid conjugate vaccine introduction - worldwide, 2018-2022. MMWR Morb Mortal Wkly Rep. (2023) 72:171–6. doi: 10.15585/mmwr.mm7207a2 PMC9949843 36795626

[2] Mogasale V Maskery B Ochiai RL Lee JS Mogasale VV Ramani E. Burden of typhoid fever in low-income and middle-income countries: a systematic, literature-based update with risk-factor adjustment. Lancet Glob Health. (2014) 2:e570–80. doi: 10.1016/S2214-109X(14)70301-8 25304633

[3] Typhoid, G.B.D. and C. Paratyphoid. The global burden of typhoid and paratyphoid fevers: a systematic analysis for the Global Burden of Disease Study 2017. Lancet Infect Dis. (2019) 19:369–81. doi: 10.1016/S1473-3099(18)30685-6 PMC6437314 30792131

[4] Robbins JD Robbins JB. Reexamination of the protective role of the capsular polysaccharide (Vi antigen) of Salmonella typhi. J Infect Dis. (1984) 150:436–49. doi: 10.1093/infdis/150.3.436 6207249

[5] Felix A Camb. R. M P B.A. A new antigen of b. typhosus: its relation to virulence and to active and passive immunisation. Lancet. (1934) 224:186–91. doi: 10.1016/S0140-6736(00)44360-6

[6] DeRoeck D Ochiai RL Yang J Anh DD Alag V Clemens JD. Typhoid vaccination: the Asian experience. Expert Rev Vaccines. (2008) 7:547–60. doi: 10.1586/14760584.7.5.547 18564010

[7] Guzman CA Borsutzky S Griot-Wenk M Metcalfe IC Pearman J Collioud A. Vaccines against typhoid fever. Vaccine. (2006) 24:3804–11. doi: 10.1016/j.vaccine.2005.07.111 16278037

[8] Mohan VK Varanasi V Singh A Pasetti MF Levine MM Venkatesan R. Safety and immunogenicity of a Vi polysaccharide-tetanus toxoid conjugate vaccine (Typbar-TCV) in healthy infants, children, and adults in typhoid endemic areas: a multicenter, 2-cohort, open-label, double-blind, randomized controlled phase 3 study. Clin Infect Dis. (2015) 61:393–402. doi: 10.1093/cid/civ295 25870324

[9] Bhutta ZA Capeding MR Bavdekar A Marchetti E Ariff S Soofi SB. Immunogenicity and safety of the Vi-CRM197 conjugate vaccine against typhoid fever in adults, children, and infants in south and southeast Asia: results from two randomised, observer-blind, age de-escalation, phase 2 trials. Lancet Infect Dis. (2014) 14:119–29. doi: 10.1016/S1473-3099(13)70241-X 24290843

[10] Szu SC Hunt S Xie G Robbins JB Schneerson R Gupta RK. A human IgG anti-Vi reference for Salmonella typhi with weight-based antibody units assigned. Vaccine. (2013) 31:1970–4. doi: 10.1016/j.vaccine.2013.02.006 PMC3839630 23422143

[11] Kumar Rai G Saluja T Chaudhary S Tamrakar D Kanodia P Giri BR. Safety and immunogenicity of the Vi-DT typhoid conjugate vaccine in healthy volunteers in Nepal: an observer-blind, active-controlled, randomised, non-inferiority, phase 3 trial. Lancet Infect Dis. (2022) 22:529–40. doi: 10.1016/S1473-3099(21)00455-2 PMC8942857 34942090

[12] Burki T. Typhoid conjugate vaccine gets WHO prequalification. Lancet Infect Dis. (2018) 18:258. doi: 10.1016/S1473-3099(18)30087-2 29485093 PMC7129039

[13] Gavi. More typhoid conjugate vaccines, more impact. (2020) GAVI. https://www.gavi.org/vaccineswork/more-typhoid-conjugate-vaccines-more-impact

[14] Take on Typhoid. A third TCV receives WHO prequalification amidst rising rates of drug resistant typhoid (2024). Available online at: https://www.coalitionagainsttyphoid.org/a-third-tcv-receives-who-prequalification-amidst-rising-rates-of-drug-resistant-typhoid/ (Accessed March 20, 2024).

[15] Kulkarni PS Potey AV Bharati S Kunhihitlu A Narasimha B Yallapa S. The safety and immunogenicity of a bivalent conjugate vaccine against Salmonella enterica Typhi and Paratyphi A in healthy Indian adults: a phase 1, randomised, active-controlled, double-blind trial. Lancet. (2023) 403(10436):1554–62. doi: 10.1016/S0140-6736(24)00249-6 38555928

[16] Martin LB Khanam F Qadri F Khalil I Sikorski MJ Baker S. Vaccine value profile for Salmonella enterica serovar Paratyphi A. Vaccine. (2023) 41 Suppl 2:S114–33. doi: 10.1016/j.vaccine.2023.01.054 37951691

[17] MacLennan CA Stanaway J Grow S Vannice K Steele AD. Salmonella combination vaccines: moving beyond typhoid. Open Forum Infect Dis. (2023) 10:S58–66. doi: 10.1093/ofid/ofad041 PMC10236507 37274529

[18] Baliban SM Allen JC Curtis B Amin MN Lees A Rao RN. Immunogenicity and induction of functional antibodies in rabbits immunized with a trivalent typhoid-invasive nontyphoidal salmonella glycoconjugate formulation. Molecules. (2018) 23. doi: 10.3390/molecules23071749 PMC6099966 30018230

[19] Skidmore PD Canals R Ramasamy MN. The iNTS-GMMA vaccine: a promising step in non-typhoidal Salmonella vaccine development. Expert Rev Vaccines. (2023) 22:918–20. doi: 10.1080/14760584.2023.2270596 37824701

[20] Rijpkema S Hockley J Logan A Rigsby P Atkinson E Jin C. Establishment of the first International Standard for human anti-typhoid capsular Vi polysaccharide IgG. Biologicals. (2018) 56:29–38. doi: 10.1016/j.biologicals.2018.09.001 30201529 PMC6238147

[21] Jin C Gibani MM Moore M Juel HB Jones E Meiring J. Efficacy and immunogenicity of a Vi-tetanus toxoid conjugate vaccine in the prevention of typhoid fever using a controlled human infection model of Salmonella Typhi: a randomised controlled, phase 2b trial. Lancet. (2017) 390:2472–80. doi: 10.1016/S0140-6736(17)32149-9 PMC5720597 28965718

[22] Micoli F Rondini S Pisoni I Proietti D Berti F Costantino P. Vi-CRM 197 as a new conjugate vaccine against Salmonella Typhi. Vaccine. (2011) 29:712–20. doi: 10.1016/j.vaccine.2010.11.022 PMC4163788 21115057

[23] Daniels EM Schneerson R Egan WM Szu SC Robbins JB. Characterization of the Salmonella paratyphi C Vi polysaccharide. Infect Immun. (1989) 57:3159–64. doi: 10.1128/iai.57.10.3159-3164.1989 PMC260784 2506132

[24] Organization, W.H. Guidelines on the quality, safety and efficacy of typhoid conjugate vaccines, 2014. (2016) World Health Organisation WHO. https://www.who.int/publications/m/item/typhoid-conjugate-vaccines-annex-3-trs-no-987.

[25] van Damme P Kafeja F Anemona A Basile V Hilbert AK Coster De I. Safety, immunogenicity and dose ranging of a new Vi-CRM(1)(9)(7) conjugate vaccine against typhoid fever: randomized clinical testing in healthy adults. PloS One. (2011) 6:e25398. doi: 10.1371/journal.pone.0025398 21980445 PMC3184126

[26] Aruta MG Lari E Simone De D Semplici B Semplici C Dale H. Characterization of enzyme-linked immunosorbent assay (ELISA) for quantification of antibodies against salmonella typhimurium and salmonella enteritidis O-antigens in human sera. Biotech (Basel). (2023) 12. doi: 10.3390/biotech12030054 PMC10443281 37606441

[27] Launay O Ndiaye AGW Conti V Loulergue P Scire AS Landre AM. Booster vaccination with GVGH shigella sonnei 1790GAHB GMMA vaccine compared to single vaccination in unvaccinated healthy European adults: results from a phase 1 clinical trial. Front Immunol. (2019) 10:335. doi: 10.3389/fimmu.2019.00335 30906291 PMC6418009

[28] Rondini S Micoli F Lanzilao L Hale C Saul AJ Martin LB. Evaluation of the immunogenicity and biological activity of the Citrobacter freundii Vi-CRM197 conjugate as a vaccine for Salmonella enterica serovar Typhi. Clin Vaccine Immunol. (2011) 18:460–8. doi: 10.1128/CVI.00387-10 PMC3067394 21248155

[29] Micoli F Bjarnarson SP Arcuri M Pind Aradottir AA Magnusdottir GJ Necchi F. Short Vi-polysaccharide abrogates T-independent immune response and hyporesponsiveness elicited by long Vi-CRM(197) conjugate vaccine. Proc Natl Acad Sci U.S.A. (2020) 117:24443–9. doi: 10.1073/pnas.2005857117 PMC7533886 32900928

[30] Gasperini G Alfini R Arato V Mancini F Aruta MG Kanvatirth P. Salmonella paratyphi A outer membrane vesicles displaying vi polysaccharide as a multivalent vaccine against enteric fever. Infect Immun. (2021) 89. doi: 10.1128/IAI.00699-20 PMC8090967 33318138

[31] Thuluva S Paradkar V Matur R Turaga K Gv SR. A multicenter, single-blind, randomized, phase-2/3 study to evaluate immunogenicity and safety of a single intramuscular dose of biological E’s Vi-capsular polysaccharide-CRM(197) conjugate typhoid vaccine (TyphiBEV(TM)) in healthy infants, children, and adults in comparison with a licensed comparator. Hum Vaccin Immunother. (2022) 18:2043103. doi: 10.1080/21645515.2022.2043103 35333702 PMC9196756

[32] Alonso-Larruga A Barrios Y Franco A Suarez-Toste I Rodriguez-Salazar MJ Matheu V. Salmonella typhi vaccination response as a tool for the stratification of risk in patients with predominantly antibody deficiencies. Diagnostics (Basel). (2022) 12. doi: 10.3390/diagnostics12102423 PMC9600355 36292112

[33] Vadrevu KM Raju D Rani S Reddy S Sarangi V Ella R. Persisting antibody responses to Vi polysaccharide-tetanus toxoid conjugate (Typbar TCV(R)) vaccine up to 7 years following primary vaccination of children < 2 years of age with, or without, a booster vaccination. Vaccine. (2021) 39:6682–90. doi: 10.1016/j.vaccine.2021.07.073 34625288

[34] Jossi SE Arcuri M Alshayea A Persaud RR Marcial-Juarez E Palmieri E. Vi polysaccharide and conjugated vaccines afford similar early, IgM or IgG-independent control of infection but boosting with conjugated Vi vaccines sustains the efficacy of immune responses. Front Immunol. (2023) 14:1139329. doi: 10.3389/fimmu.2023.1139329 37033932 PMC10076549

[35] Oldrini D Benedetto Di R Carducci M Simone De D Massai L Alfini R. Testing a recombinant form of tetanus toxoid as a carrier protein for glycoconjugate vaccines. Vaccines (Basel). (2023) 11. doi: 10.3390/vaccines11121770 PMC10747096 38140177

[36] Rigsby P Beamish E Hockley J Atkinson E Hitri K Jones E. Evaluation of a standardised Vi poly-l-lysine ELISA for serology of Vi capsular polysaccharide antibodies. Biologicals. (2020) 66:21–9. doi: 10.1016/j.biologicals.2020.05.002 PMC7391004 32571662

